# Studying politics at the local level in Germany: a tale of missing data

**DOI:** 10.1007/s12286-022-00551-7

**Published:** 2023-01-16

**Authors:** Klaudia Wegschaider, Martin Gross, Sophia Schmid

**Affiliations:** 1 University of Oxford, Oxford, UK; 2 Ludwig Maximilian University of Munich, Munich, Germany

**Keywords:** Manifestos, Local politics, Party competition, Municipalities, Voting age

## Abstract

What are the strategies of political parties in multi-level government systems and how do voters respond to these strategies? At the national and state level, many researchers have worked with data from party manifestos, candidate lists, party press releases, parliamentary questions, and parliamentary speeches. At the local level, these standard components of the political science toolbox are not available to researchers in many countries—including Germany. The absence of these data means that politics at the local level remains chronically understudied. In this research note, we draw attention to the dearth of data when studying local politics and the questions that we cannot answer as a result. Specifically, we document our failed attempt to collect local party manifestos in smaller municipalities which would have been the basis for an analysis of political parties’ strategies in response to the lowering of voting ages in local elections in German states. We point to examples from other countries that show that it does not have to be this way.

## Introduction

What are the strategies of political Parties In multi-level government systems and how do voters respond to these strategies? These are fundamental questions for our understanding of political processes in modern representative democracies. Since the “methodological nationalism” in comparative politics and in the study of political parties’ strategies still prevails (Jeffery und Schakel 2013), it comes as no surprise that many scholars have worked with national level-data from party manifestos (e.g. Adams et al. 2004, 2014), candidate lists (e.g. Buisseret et al. 2022; Däubler et al. 2021), party press releases (e.g. Debus und Florczak 2022; Ennser-Jedenastik et al. 2022), parliamentary questions (e.g. Höhmann und Krauss 2022; Martin 2011), or parliamentary speeches (e.g. Bäck et al. 2021; Proksch et al. 2019) to address questions of political party behaviour. For Germany, the case we are focusing on here in this research note, scholars “scaled down” (Snyder 2001) and also turned their eyes to the state level—the German *Länder*—to investigate political parties’ strategies and the responses of voters. While the regional level continues to gain more legal, economic and financial competencies in European democracies (see Hooghe et al. 2010, 2016), it is at the *local* level where citizens get in touch immediately with political actors and institutions.

At the local level, the standard components of the political science toolbox are not available—neither for Germany nor many other countries. Minutes of local councils are, if at all, often only available in reduced or bullet point form due to the resources involved in transcription. But other documents—such as party manifestos, candidate lists, parliamentary questions, distribution of portfolios, and press releases—do not need to be created, they need to be systematically collected. Currently, a researcher wishing to work with such material across different local governments stands before an arduous and often fruitless data collection effort. The loss of these data means that politics at the local level remains chronically understudied.

Even though there are studies dealing with potential party-related factors impacting local policy-making (see Sect. 2), they usually have at least one of the following limitations. First, they either focus on large municipalities, usually those with more than 100,000 inhabitants, or on the German counties, the so-called *Landkreise*, thus only studying a small section of political and voter behaviour. Second, if the policy positions of parties are relevant for the explanation of local politics, most of these studies use party positions from federal or state party organizations instead of local party positions, thus not taking into account potential policy position variations within party organizations and between parties in different municipalities. Third, analysing individual-level data, for instance citizens’ voting behaviour in local elections is probably the biggest lacunae in German local politics research. Even investigating the impact of a change in local electoral laws—impacting the eligibility of citizens to vote—on party behaviour turned out to be an impossible task.

In this research note, we draw attention to the dearth of data when studying local politics and the questions that we cannot answer as a result. Specifically, we document our failed attempt to collect local party manifestos in smaller municipalities. We will describe our attempt to systematically analyse the impact of lowering citizens’ voting age in local elections in several German states from 18 to 16 on local parties’ behaviour. In doing so, we will address the challenges and problems regarding data collection, and we discuss potential ways to improve the availability of party- and individual-level data at the local level in Germany. As examples from other countries show, it does not have to be this way.

## Why it matters: the importance of studying local democracy

While there are many parallels, the dynamics of local politics cannot simply be equated with the ones at the national level. At the local level, the political landscape can be completely different. Parts of the country have locally specific parties and, sometimes, major national parties do not even compete because of the strong electoral performance of independent local lists (Göhlert et al. 2008; Jankowski et al. 2022). Therefore, one cannot assume that findings at the national level simply translate to the subnational level, even though the patterns of local party competition and government formation in large cities in Germany are similar to the ones at the federal and state level (Debus and Gross 2016; Gross 2018, 2023; Gross and Debus 2018a; Gross and Jankowski 2020). The question arises, however, to what extent national dynamics can be observed in small localities where voters and politicians are more likely to know each other personally.

Given that local governments, particularly local administrations in Germany, manage important policy areas (such as childcare, public transit, and levying local taxes, fees and charges, to name a few) and act as executors of federal and state laws (Benz and Zimmer 2011; Gross and Krauss 2023), it is surprising how little we know about local politicians’ preferences and how they shape their policy-making decisions. Only a small number of studies exist that identify how political preferences of local politicians impact local policy positions and policy output, for example with regard to registration fees for same-sex unions (Debus et al. 2013), local tax rates (Person 2020), partisan positions on electricity grid construction (Fink et al. 2022). It can also be questioned whether local party differences play a role in the decision of joining climate networks (Schulze and Schoenefeld 2022), or the effect of the electoral cycle on public administration decisions (Garmann 2017). These studies usually rely either on county-level policy positions of local parties or use party dummies to assess party differences in local policy-making, thus disregarding the variation both within parties across localities and between established parties and their local-level competitors (Gross und Jankowski 2020; Jankowski et al. 2022).

Therefore, especially in Germany, the study of local politics holds largely untapped potential. Due to its federal structure, there is significant variation in terms of institutional design at the local level. To name just one example—which inspired our initial research idea—some of the German states have already lowered the voting age to sixteen at the local level. Unlike studies at the national level, analyses at the subnational level benefit from the larger number of units that can be observed. Data that grants insights into the actions of parties and voters at the local level—such as election manifestos—can open the door to a better understanding of democracy on a small scale.

## Studying voting age sixteen in Germany

Voting at sixteen has been a recurring topic in German politics. Since the late 1990s, eleven German *Länder *have lowered the voting age to sixteen (Leininger and Faas 2020). So far, there has been limited scholarly attention to the effects of decreasing the voting age. The few studies that consider the impact of youth enfranchisement have looked at other countries and, mostly, at the effect on turnout (Zeglovits and Aichholzer 2014; Franklin 2020), political efficacy (Leininger et al. 2022), satisfaction with democracy (Sanhueza Petrarca 2020), or a combination thereof (Aichholzer and Kritzinger 2020). What remains unexplored is how lowering the voting age has affected party strategies.

This idea led to the question that became the starting point for our research: does youth enfranchisement lead to a better representation of youth interests at the local level? Since the hypothesized mechanism is electoral, party manifestos ahead of local elections are well-suited to address this question. The plan was to analyse whether the coverage of topics relevant to youth voters increases in local manifestos after enfranchisement. In other words, when reviewing the manifestos in detail, we ask: are there policies aimed specifically at young voters? The aim was to code the occurrence of these policies in manifestos from elections both before and after enfranchisement in a set of German *Länder*.

Youth enfranchisement in Germany is especially well-suited for this project, not only because of the subnational variation in enfranchisement, but also because of the low level of vertical party integration. It is analytically useful that the voting age at the municipal level is decided at the *Länder* level; this means that the level that decides on enfranchisement is not the level that faces the potential direct electoral consequences of this decision. In other words, the politicians that decided on the lowering of the voting age are not the same politicians that have to decide whether and how to react to the new voting age threshold in their local campaigns. Prior research on local manifestos has shown that there is no or very low vertical party coordination of manifestos (see Gross and Jankowski 2020); this means that the *Land* parties do not set the manifestos for municipal parties, as is the case with framework manifestos in Belgium or Spain, respectively (see van de Voorde et al. 2018).

The research design envisioned paired comparisons between municipalities in *Länder* that had and had not lowered the voting age. We anticipated that it would be more challenging to collect manifestos from long ago, and therefore focused on the states that lowered the voting age more recently, while excluding the earlier cases of enfranchisement. From the *Länder *that have already lowered the voting age to 16, we selected the following: Saxony-Anhalt (lowered the voting age to 16 in 2010), Brandenburg (lowered the voting age to 16 in 2011), and Baden-Wuerttemberg (lowered the voting age to 16 in 2013). These were paired with Saxony, Mecklenburg-Western Pomerania, and Bavaria, respectively. To the extent possible, these pairings were supposed to follow the most-similar logic. The paired *Länder* all had local elections at roughly similar times, both before and after enfranchisement (see Table 1).

**Table 1:** Detailed Case Selection. (Source: Own illustration)

*Länder* with voting age at 16			*Länder* without voting age at 16		
*Land*	Local election before voting age at 16	Local election after voting age at 16	*Land*	Local election	Local election
Saxony-Anhalt	2009	2014	Saxony	2009	2014
Brandenburg	2008	2014	Mecklenburg Western-Pomerania	2009	2014
Baden-Wuerttemberg	2009	2014	Bavaria	2008	2014

Saxony-Anhalt lowered the voting age in 2010, Brandenburg in 2011, and Baden-Wuerttemberg in 2013

We followed a systematic matching procedure of the municipalities, based on population size and median age. Both indicators were taken from the scientific use file of the *Wegweiser Kommune*, provided by the *Zentrum für Interdisziplinäre Regionalforschung* and the *Bertelsmann Stiftung* (Zentrum für Interdisziplinäre Regionalforschung und Bertelsmann Stiftung 2022). The first step involved selecting ten municipalities (including *Gemeinde* and *kreisfreie Stadt*, excluding *Landkreis*) from each *Land* that had lowered the voting age. For this, we divided the full sample of municipalities into four groups based on population size: (a) small (≥ 10,000 to < 20,000 inhabitants), (b) medium-small (≥ 20,000 to < 50,000 inhabitants), (c) medium-large (≥ 50,000 to < 100,000 inhabitants), and (d) large (≥ 100,000 inhabitants). We excluded municipalities with less than 10,000 inhabitants because we anticipated that data collection would be especially challenging in very small municipalities.

For each *Land*, we selected a set of large, medium-large, medium-small, and small municipalities.^1^ Technically, the idea was to use a random stratified sampling. In reality, however, some strata only consisted of one or two cases in the first place (for example, there is only one large municipality in our large population range in Brandenburg (that is, Potsdam) because Cottbus is no longer a so-called *Großstadt* with at least 100,000 inhabitants). As part of the second step, we matched municipalities within the same population stratum based on median age. Ideally, we would have used the share of citizens in the 16–17 age range, but this data was not available. As stated before, municipalities were matched across *Länder* based on the most similar *Länder* pairings (see Table 1). In the end, we had six *Länder*, two elections, ten municipalities, and roughly five parties per election and municipality—theoretically amounting to an estimated 600 manifestos that we hoped to collect (see Table 2).

Note, however, that the actual number of potential manifestos to be collected would have been lower because especially in less populated German *Länder* (like Mecklenburg Western-Pomerania) our matching procedure produced several double entries of best-matched municipalities based on population and median age. This is particularly the case when looking at our backup (second match) municipalities. We had generated these second matches as backup options from the beginning on in case we ran into some difficulties with data collection in our first matches. Yet, even these backup options did not solve our problems with the serious lack of data on local party manifestos (see Sect. 4).

**Table 2:** Matched municipalities across six German states. (Source: Own illustration)

Municipalities in states with voting age at 16				Municipalities in states without voting age at 16 (first match)				Municipalities in states without voting age at 16 (second match)			
Municipality	State	Pop	Age	Municipality	State	Pop	Age	Municipality	State	Pop	Age
Uhingen	BW	13,963	45.2	Weißenhorn	BY	13,268	45.2	Grafing bei München	BY	13,369	45.4
Tettnang	BW	18,473	45.5	Marktoberdorf	BY	18,170	45.5	Mühldorf am Inn	BY	18,305	45.6
Weil am Rhein	BW	29,683	44.8	Neuburg a. d. Donau	BY	28,910	44.0	Landsberg am Lech	BY	28,432	44.1
Bad Rappenau	BW	20,268	46.6	Traunreut	BY	20,537	46.5	Gauting	BY	20,158	46.4
Fellbach	BW	44,611	44.5	Memmingen	BY	42,201	45.2	Straubing	BY	46,027	45.7
Esslingen am Neckar	BW	90,378	43.3	Bayreuth	BY	71,601	43.6	Rosenheim	BY	60,889	43.2
Ludwigsburg	BW	91,116	42.1	Bamberg	BY	71,952	42.9	Neu-Ulm	BY	55,689	42.5
Konstanz	BW	81,692	39.5	Erlangen	BY	106,423	40.4	Freising	BY	45,857	39.5
Ulm	BW	120,714	40.8	Würzburg	BY	124,219	40.2	Ingolstadt	BY	131,002	41.7
Reutlingen	BW	112,452	44.2	Fürth	BY	121,519	43.5	Landshut	BY	67,509	44.4
Genthin	ST	14,466	52.2	Meerane	SN	14,850	52.6	Löbau	SN	15,288	51.9
Braunsbedra	ST	11,285	49.2	Nossen	SN	10,851	49.7	Bannewitz	SN	10,480	48.7
Merseburg	ST	33,317	49.9	Bautzen	SN	39,475	50.0	Meißen	SN	27,273	50.1
Quedlinburg	ST	24,742	51.5	Glauchau	SN	23,231	51.6	Zittau	SN	25,792	52.0
Salzwedel	ST	24,084	50.0	Limbach-Oberfrohna	SN	24,014	50.2	Delitzsch	SN	24,911	50.4
Stendal	ST	40,079	48.4	Freital	SN	39,547	48.7	Freiberg	SN	40,829	47.9
Naumburg (Saale)	ST	32,756	51.1	Pirna	SN	37,768	51.3	Grimma	SN	28,411	50.5
Dessau-Roßlau	ST	83,061	52.5	Zwickau	SN	91,066	50.4	Plauen	SN	64,077	50.4
Magdeburg	ST	232,306	46.3	Chemnitz	SN	243,521	48.8	Wilsdruff	SN	13,701	46.3
Halle (Saale)	ST	232,470	45.3	Chemnitz	SN	243,521	48.8	Wilsdruff	SN	13,701	46.3
Oberkrämer	BB	10,603	47.9	Boizenburg/Elbe	MV	10,350	48.7	Hagenow	MV	11,443	47.6
Pritzwalk	BB	11,909	51.3	Demmin	MV	11,342	52.0	Wolgast	MV	12,273	50.2
Hennigsdorf	BB	25,928	49.4	Güstrow	MV	28,791	49.9	Neustrelitz	MV	20,476	50.6
Strausberg	BB	25,946	49.6	Güstrow	MV	28,791	49.9	Neustrelitz	MV	20,476	50.6
Rathenow	BB	24,127	51.8	Waren (Müritz)	MV	21,042	50.8	Neustrelitz	MV	20,476	50.6
Bernau bei Berlin	BB	36,547	48.1	Wismar	MV	42,392	48.6	Neustrelitz	MV	20,476	50.6
Cottbus	BB	99,491	48.5	Schwerin	MV	92,138	48.7	Ludwigslust	MV	12,243	49.1
Frankfurt (Oder)	BB	57,649	50.0	Stralsund	MV	57,525	48.3	Neustrelitz	MV	20,476	50.6
Brandenburg an der Havel	BB	71,032	50.5	Neubrandenburg	MV	63,311	49.1	Neustrelitz	MV	20,476	50.6
Potsdam	BB	164,042	42.3	Rostock	MV	204,167	45.1	Greifswald	MV	56,685	39.3

Population (Pop.) is measured in number of inhabitants

Median age (Age) is measured in years

State abbreviations: *BB* Brandenburg, *BW* Baden-Wuerttemberg, *BY* Bavaria, *MW* Mecklenburg Western-Pomerania, *SN* Saxony, *ST* Saxony-Anhalt

In terms of analysis of the manifestos, the plan was to proceed iteratively. In the process of reading the manifestos, we would have marked the language and policies that appear to target youth voters specifically. In terms of policy, an example could be local youth centres. In terms of language, there may have been a specific section on young people in the city. This would have been the basis for simple text analysis based on the manifestos, such as word frequency. Here, we would have compared whether there is an observable increase in the appeals to and targeting of youth from the pre-enfranchisement to the post-enfranchisement election in the treated *Länder*. We would have then checked whether we could observe a similar change in the untreated matched *Länder*. Overall, while informed by causal inference considerations, the nature of the analysis was meant to be primarily exploratory.

Even beyond this project which attempts to deal with the impact of voting age on party strategies, the manifestos could have shed light on the dynamics of representation and party competition at the municipal level. These include, but are not limited to the following: To what extent do the issue dimensions that shape competition at the national level also surface in local politics? Have local politics addressed the need for action on preventing and adapting to the climate catastrophe to the political agenda at the municipal level? Whose preferences are best represented at the local level?

Beyond manifestos, data on local parties could serve as important control variables in other projects. For example, an important question in political science is how social movements shape political behaviour. One may ask how the protests against COVID-19 measures shaped support for far-right parties (cf. Heinze and Weisskircher 2022). To study this, researchers may rely on geocoded data of local protests and the vote share of a far-right party in national elections. If the results show a positive correlation, the question arises whether local protests are themselves an outcome of stronger party organisation at that same locality and whether this issue has been included in local party manifestos. After all, the emergence of protests is unlikely to be random. To control for this, however, researchers need reliable information on local party politics, such as membership numbers. This shows that data on local party politics is important, even in cases where local party politics is neither the dependent nor the independent variable in a research project.

## The (failed) attempt to collect local manifestos in smaller municipalities

Our starting point was using existing data from the *Local Manifesto Project* (Gross and Jankowski 2020), which already contained more than 1,000 election manifestos from parties competing in German municipalities. Many of these, however, were from *Länder* that were not part of our research design, and they were (almost) exclusively available for cities with more than 100,000 inhabitants.

Even though we had already prepared ‘backup’ municipalities (second matches in Table 2) because we expected some complications in data collection, the actual data collection process proved to be more complicated than even our worst expectations. The data collection mostly took place in the fall of 2021. The first challenge was to identify which parties had competed in specific local elections. We anticipated a low data availability in small municipalities, as often some national-level parties do not compete in specific localities. In several municipalities, only the Social Democratic Party of Germany (SPD), the Christian Democratic Union of Germany (CDU) (or the Christian Social Union in Bavaria [CSU]), and a local list had stood for election in the first place. This type of missing data from other parties, however, would not have been problematic because it also tells us something about the structure of local party competition in such municipalities.

The second challenge was to collect the manifestos of the parties that ran for local elections in these municipalities. Yet, in small municipalities, some parties did not always have a local chapter. This applied particularly to the Greens, the Free Democratic Party (FDP), and The Left. Instead, these parties are frequently organized at the county level (*Kreisebene*) and it was not always clear—online or even for the contact persons that we had reached—which municipality belonged to which specific county-level party organization. Apart from complicating our correspondence, this structure also meant that one manifesto was written for several smaller municipalities within that *Kreis*, meaning that our observed signal of party responsiveness, the local manifestos, could not be uniquely matched to our units of observation, the specific municipalities. While this undermined our original paired design, the *Kreis* is still not equivalent with the *Land*, where enfranchisement was passed. It therefore still holds that the party level confronted with the reality of the new voting age (the municipality or *Kreis*) was separate from the party level at which enfranchisement had been decided (the *Land* level).

Once a local contact could be identified, the main barrier was low responsiveness. It quickly crystallized that responsiveness via email was low and we turned to the phone instead. The aim was to get information about the availability of the election manifestos without much delay. That said, the relevant phone numbers were often not available online. When parties had local offices, they were rarely staffed and difficult to reach by phone. If phone numbers were available, the persons in charge published their private phone numbers.

When we were able to reach someone from the local party, the relevant manifestos had frequently been lost. Only in a few cases was it possible to download the manifestos and electoral flyers of previous local elections directly from local parties’ websites. We had already anticipated that there would hardly be any digital documents from the year 2008/2009. But even for municipal elections from the year 2019 (outside of our sample), the documents are not available online or in digital format. Older election manifestos and flyers, although they had once existed in digital form for printing, had often not been stored. Printed leaflets that are left over at the end of the election campaign are usually destroyed instead of archived. They are also often a casualty of turnover when the individuals who collected the manifestos leave their positions—or their position is altogether not refilled. If files had not been deleted and printouts not destroyed, this was due to the exceptional efforts of individuals. In one case, and one case only, a local party representative had kept the manifestos of all competing parties of the last twenty years. Where local manifestos had survived, they were generally only available as physical copies. Not only did this involve the task of searching for the manifestos but also scanning them—we are especially grateful to all those who had helped us with this task.

In the end, we managed to collect 26 manifestos out of an estimated 150 manifestos (see Table 3). This was far from the approximately 600 manifestos that we had intended to collect. It was clear relatively quickly that it would not be possible to obtain the necessary amount of data for even an exploratory analysis. Even in larger cities (≥ 100,000 inhabitants), it was not possible to obtain the manifestos of all parties that had competed in local elections. The data in Table 3 even paint a sadder picture: in 110 out of 150 cases (73%) we did not even get a response from party representatives when we requested their local election manifestos. Only in 26 out of 150 cases could parties provide us with local election manifestos (17%). Nonetheless, this effort was not in vain. The collected manifestos will be available as part of the *Local Manifesto Project* database and the experience of this failed data collection has brought to light a need to address the lack of data on local politics.

**Table 3:** Data availability of local party manifestos in municipalities. (Source: Own illustration)

Population size of municipalities	Manifestos			
	Available	No response	Not available	Sum
*<* *25,000 inhabitants*	6	35	9	50
CDU	2	7	1	–
SPD	2	7	1	–
Greens	0	9	1	–
FDP	1	7	2	–
The Left	1	5	4	–
*25,000* *>* *x* *>* *100,000 inhabitants*	12	58	5	75
CDU	2	13	0	–
SPD	2	12	1	–
Greens	5	10	0	–
FDP	1	12	2	–
The Left	2	11	2	–
*>* *100,000 inhabitants*	8	17	0	25
CDU	2	3	0	–
SPD	2	3	0	–
Greens	2	3	0	–
FDP	1	4	0	–
The Left	1	4	0	–
Sum	26	110	14	150

## Toward transparent and accessible local party manifesto collection and storage

It is evident that the current system—which is completely dependent on the discretion of parties and the diligence of individuals to ensure that local manifestos are collected and shared—is not working satisfactorily, neither for scholars nor for citizens interested in local politics. So, what are some better ways of data collection and storage to make local politics more transparent?

Parties can play an important role in preserving manifestos, but this responsibility should not rest with them alone. At the federal level, it is party-affiliated archives and party-affiliated foundations that collect manifestos. In the case of the Christian Democrats, even the manifestos of the *Länder* level are available going back many decades. While this is commendable, it should not be up to parties’ goodwill as to whether manifestos are collected or not. And as our data collection effort shows, this system fails at the local level. While local manifestos are often lost because their value for parties may not be evident after an election, there can also be strategic reasons to make them unavailable. In some cases, as had been brought to our attention in several phone calls, parties did not want it to be possible to determine what positions they held in the past. In other words, parties face a conflict of interest because they may have incentives to ‘lose’ or alter the data. This decreases voters’ ability to hold parties accountable based on their electoral pledges and hinders research on local politics.

To avoid conflicts of interest and to systematize the data, other actors should spearhead the collection of manifestos. Researchers have already stepped in to fill this lacuna. At the national level, the *Manifesto Project Database* has been a valuable source on party pledges across countries (see, e.g., Volkens et al. 2020). Along with election manifestos for many European democracies, federal and regional election manifestos of German parties are made publicly available by the *Political Documents Archive* (see Benoit et al. 2009; Gross and Debus 2018b). At the local level, there has also been progress on collecting and sharing local manifestos. Regarding the largest municipalities in Germany with more than 100,000 inhabitants, at least the collection and storage of local party manifestos (and local coalition agreements) for the most recent local elections—and for the large cities in Hesse and North Rhine-Westphalia even going back until the early 1990s—have been secured via the *Local Manifesto Project* (see Gross and Jankowski 2020). Yet, this data collection effort was only possible because of a PhD project (Gross 2016) and an externally funded project on local election results in the German state of Lower Saxony (Jankowski et al. 2019), where a couple of student assistants—and even students in their own free time—helped in collecting the manifestos. Even though to some degree older local election manifestos can be found via archived versions of municipal and party websites, this is not doable for smaller municipalities. As outlined above, local party organization in smaller municipalities is not as professionalized as in larger cities, and sometimes local parties do not even have websites. Accordingly, this effort alone is not sufficient to make local party manifestos available to the scientific community in a comparative and extensive way.

One example from The Netherlands shows that it does not have to be this way. Until recently, as in Germany, local party manifestos also had to be collected with the help of a large number of student assistants and even local media partners (see Otjes 2021). Yet, the situation in The Netherlands changed dramatically and should now be seen as a role model for data collection and storage regarding local political actors’ behavior in Germany. Due to the efforts of the Open State Foundation and partners (Open State Foundation 2022), local election manifestos and even local coalition agreements can be found online for all Dutch municipalities for the two most recent legislative periods at the local level (2014–2018 and 2018–2022). Furthermore, the Open State Foundation successfully urged Dutch municipal councils to provide all council-related information (such as voting records, bills, or parliamentary questions) in a standardized and structured machine-readable format. This opens exciting new ways for gaining deeper insights into Dutch local politics (see, e.g., Otjes et al. 2022). In Germany, however, we are only scratching the surface of this data treasure—and, again, only if a large amount of external funding is provided.^2^

Another option would be to create an official channel to record the political aims made ahead of elections. In this regard, the transparency laws in several Latin American countries can serve as a source of inspiration. Since political parties tend to be less coherent in several Latin American countries, party manifestos have lower importance. However, in some countries, political candidates—also at the local level—are required to officially submit their programmes. In Colombia, for example, when candidates register to run for the office of mayor, they must submit a programme (*programa de gobierno*, comparable to a manifesto).^3^ It is then understood that the elected candidate has the mandate to pursue this programme. A similar provision exists in Ecuador where candidates must share their *plan de trabajo* at the time of registering. This information is generally collected and made available through the relevant electoral authority. While this is not quite the same as a party manifesto, the idea of having one point of information that formally collects commitments made ahead of elections can nonetheless be applied to the context of party manifestos. We encourage lawmakers to follow such good practices.

## Conclusion

In this research note, we have drawn attention to the lack of data on local party politics. Many types of data that form part of the standard toolkit of political science research at the national or regional levels are not available at the local level. This includes, among other things, local party manifestos. We document our unsuccessful efforts to collect local party manifestos from small to large localities in Germany. In the process, we ran into a series of logistical and political obstacles that meant that most local manifestos were not available. In almost three-quarter of all inquiries, local parties did not even respond to our request.

This lack of election- and party competition-related data at the local level implies that many research questions cannot be answered. In our case, we were not able to find out whether or how local parties responded to the lowering of the voting age to 16. We conclude that we cannot rely on political parties to collect their own manifestos. Instead, we should equip researchers or research organisations with the resources necessary to gather the data (as has been done in The Netherlands) or create official channels by which manifestos are collected and shared (similar to the situation in Colombia or Ecuador). Third-party data collection circumvents some of the conflicts of interest that parties may face with regard to preserving and sharing their manifestos.

While our focus has been on highlighting the importance of these data for research purposes, the unavailability of local manifestos also points to other challenges of democratic accountability. It ought to be easy for voters to consult previous election manifestos. Without this transparency, accountability suffers. This is especially true at the local level. At higher levels of government or in large cities, the media is likely to report on the promises and actions of parties and politicians. Even when manifestos are not available, it is possible to fall back on other sources. In the case of smaller municipalities, or larger municipalities that are not covered prominently in regional newspapers, this information often just exists in the realm of memory. In the short run, the lack of data renders local politics non-transparent. In the medium and long run, we are also losing a treasure of local histories if we do not look for research strategies such as those mentioned above that could reduce this loss. We would be pleased if other research approaches could show new perspectives here.

## Footnotes

1 Since the local population sizes vary across *Länder*, we did not use the same intervals for all of them. For *Baden-Wuerttemberg*, we selected two small, three medium-small, three medium-large, and two large municipalities. For *Saxony-Anhalt*, we selected two small, five medium-small, one medium-large, and two large municipalities. For *Brandenburg*, we selected two small, four medium-small, three medium-large, and one large municipality.

2 For instance, this is the case for the most recent local elections in Bavaria and North Rhine-Westphalia in 2020, where even socio-demographic characteristics of candidates running for local councils and more fine-grained data on local election results for the *Kreistage* has been made publicly available (see Pollex et al. 2021; Nyhuis et al. 2022).

3 See Article 259 of the Political Constitution of Colombia (https://pdba.georgetown.edu/Constitutions/Colombia/colombia91.pdf) as well as Article 1 of the Law 131 of 1994 (https://www.funcionpublica.gov.co/eva/gestornormativo/norma.php?i=4818#:~:text=%22Por%20la%20cual%20se%20reglamenta,y%20se%20dictan%20otras%20disposiciones%22.&text=ARTICULO%202%C2%BA%2D%20En%20desarrollo%20de,los%20t%C3%A9rminos%20de%20esta%20ley).
